# Interdialytic weight gain of less than 2.5% seems to limit cardiac damage during hemodialysis

**DOI:** 10.1177/0391398820981385

**Published:** 2020-12-18

**Authors:** Junko Goto, Ulf Forsberg, Per Jonsson, Kenichi Matsuda, Bo Nilsson, Kristina Nilsson Ekdahl, Michael Y Henein, Bernd G Stegmayr

**Affiliations:** 1 Institute of Public Health and Clinical Medicine, Division of Medicine, Umeå University, Umeå, Sweden; 2 Department of Emergency and Critical Care Medicine, School of Medicine, University of Yamanashi, Yamanashi, Japan; 3 Department of Internal Medicine, Skellefteå County Hospital, Skellefteå, Sweden; 4 Department of Immunology, Genetics and Pathology, Uppsala University, Uppsala, Sweden; 5 Linnaeus Centre of Biomaterials Chemistry, Linnaeus University, Kalmar, Sweden

**Keywords:** Biocompatibility, emboli, hemodialysis, heart, interdialytic weight gain, troponin, NT-pro-BNP, pentraxin

## Abstract

**Aims:**

To investigate if a single low-flux HD induces a rise in cardiac biomarkers and if a change in clinical approach may limit such mechanism.

**Material and methods:**

A total of 20 chronic HD patients each underwent three different study-dialyses. Dialyzers (low-flux polysulfone, 1.8 sqm) had been stored either dry or wet (Wet) and the blood level in the venous chamber kept low or high. Laboratory results were measured at baseline, 30 and 180 min, adjusted for the effect of fluid shift. Ultrasound measured microemboli signals (MES) within the return line.

**Results:**

Hemodialysis raised cardiac biomarkers (*p* < 0.001): Pentraxin 3 (PTX) at 30 min (by 22%) and at 180 min PTX (53%), Pro-BNP (15%), and TnT (5%), similarly for all three HD modes. Baseline values of Pro-BNP correlated with TnT (rho = 0.38, *p* = 0.004) and PTX (rho = 0.52, *p* < 0.001). The changes from pre- to 180 min of HD (*delta*-) were related to baseline values (Pro-BNP: rho = 0.91, *p* < 0.001; TnT: rho = 0.41, *p* = 0.001; PTX: rho = 0.29, *p* = 0.027). *Delta* Pro-BNP (rho = 0.67, *p* < 0.001) and TnT (rho = 0.38, *p* = 0.004) correlated with inter-dialytic-weight-gain (IDWG). Biomarkers behaved similarly between the HD modes. The least negative impact was with an IDWG ⩽ 2.5%. Multiple regression analyses of the Wet-High mode does not exclude a relation between increased exposure of MES and factors such as release of Pro-BNP.

**Conclusion:**

Hemodialysis, independent of type of dialyzer storage, was associated with raised cardiac biomarkers, more profoundly in patients with higher pre-dialysis values and IDWG. A limitation in IDWG to <2.5% and prolonged ultrafiltration time may limit cardiac strain during HD, especially in patients with cardiovascular risk.

## Introduction

Cardiovascular (CV) morbidities and mortalities represent the prime complications in patients undergoing chronic hemodialysis (HD).^1^ In these patients, mortality is generally related to raised pre dialysis drawn cardiac markers such as NT-pro-BNP (Pro-BNP) and troponin T (TnT).^2–4^ While Pro-BNP is secreted in response to volume and pressure overload, TnT is released in response to myocardial injury.^5^ Also pentraxin 3, is considered as a marker of myocardial ischemia,^6,7^ that is released by cardiac myocytes through the inflammation pathway,^8,9^ but also by activated leukocytes.^10^

Since these markers of cardiac function are partly cleared by the kidneys, their levels are usually raised when kidney failure worsens and patients are put on hemodialysis.^1,6,11–13^ When urine output ceases fluid is retained in the body due to intake of food and fluids and has to be removed by ultrafiltration during HD to avoid death by pulmonary edema and subsequent heart failure. A larger proportion of retained fluid between dialyses, the inter-dialytic-weight-gain (IDWG), is related to cardiac decompensation and increased mortality.^14–16^ Cardiac biomarkers NT-pro-BNP (Pro-BNP)^17–19^ and TnT^19^ levels (correction for fluid shifts during HD), have been reported to increase with low-flux dialyzers and decrease with high-flux HD and especially hemodiafiltration (HDF). The decrease in markers during high-flux HD is related to a higher clearance of the molecules through those dialyzers.^19^

The HD procedure previously caused considerable interactions between blood and the cellulose or synthetic dialyzer membrane in the extracorporeal circuit. This caused activation of the complement system, platelets, leukocytes and coagulation.^20,21^ Such interaction is considered negligible when using modern synthetic membranes made of polysulfone and polycarbonate.^20,22^ The extracorporeal circuit also contains air in venous chambers (air traps) and dry stored dialyzers.^23^ The shape and blood level (low or high) in the air trap of the venous chamber enables more or less micro-air bubbles to return to the patient.^24^ In vitro studies indicate that wet stored dialyzers limit such air contamination.^25^ Such air may activate clotting^26^ and also return back into the blood lines of the patient. Numerous air bubbles are not fully absorbed but covered by fibrin and deposit as microemboli in the body. The first location to be trapped are the lungs^27^ but passage into the arterial circuit have verified such emboli also in the brain and heart.^28^ There is, to our knowledge, no comparative study that clarifies if modern polysulfone dialyzers cause different biochemical responses in different dialysis settings using either “high” versus “low” blood levels in the air trap and steam sterilized dry dialyzers or wet stored gamma irradiated dialyzers. Do such variables alter inflammatory and especially cardiac biomarkers differently during HD.

The aim of this study was to investigate if a single low-flux HD session induces a rise in cardiac biomarkers and if so can a change in clinical approach limit such mechanism.

## Materials and methods

### Study population

The prospective study included data from 60 HD sessions performed by 20 (8 women and 12 men) long-term chronic HD patients who served as their own controls in cross-over settings. They were all recruited from the same center, Umea University Hospital, Sweden. All patients were in stable condition without infections, embolic events or active tumors. The study complies with the Declaration of Helsinki, and the locally appointed ethics committee had approved the research protocol (EPN 05-138M, addition date 20071016; EPN2012-42-31M, 20120306). Informed consent was obtained from the patients. Women didn’t differ from men in age (65 ± 12 years vs 66 ± 10 years) or duration of HD (vintage time: 33 ± 28 months vs 43 ± 27 months, respectively). The primary diagnosis causing end stage renal disease was undefined chronic renal failure (*n* = 7), polycystic kidney disease (*n* = 3), diabetic nephropathy (*n* = 3), glomerulonephritis (*n* = 3), pyelonephritis (*n* = 2), and hypertension (*n* = 2). Vascular access used was central venous catheter (*n* = 10), arteriovenous fistulae (*n* = 7), and AV-graft (*n* = 3). More extensive demographic data of these patients are presented in Table 1 and were also presented in a previous study focusing on distribution of microemboli of air.^29^ In this study we extended and included analyses of cardiac and biomarkers.

**Table 1.** shows demographic data including mean values and ±SD of numeric data achieved at baseline of HD of the study series. The QRS duration (measured by ECG) and ejection fraction (echocardiography/magnetic resonance imaging), closest in time to the study (in general within ±6 months) are given as well as the Charlson comorbidity score (grade 0–30) and the history of number of cardiovascular areas involved (myocardial infarction, congestive heart failure, stroke, peripheral vascular disease).

Patient code	Gender	Age (years)	DM	Dry weight (kg)	IDWG (%)	BP_syst_ ± SD (mmHg)	BP_diast_ ± SD (mmHg)	Pulse ± SD (beats/min)	QRS-dur. (ms)	Ejection fraction (%)	Charlson score	CV score	ProBNP ± SD	Troponin T ± SD	Pentraxin ± SD
1	1	55	0	53.2	1.7	154 ± 9.0	85 ± 3.8	62 ± 1.2	94.0	56.0	2.0	0.0	3161 ± 1299	32 ± 0	8.4 ± 1.8
2	2	77	0	80	2.5	143 ± 29.5	68 ± 11.6	64 ± 1.5	98.0	50.0	5.0	0.0	6286 ± 820	407 ± 19	4.4 ± 0.7
3	2	61	0	138	1.8	142 ± 17	78 ± 6.2	52 ± 2.1	111.0	65.0	2.0	0.0	1563 ± 125	51 ± 1	2.5 ± 0.3
4	2	72	0	82	0	136 ± 18	83 ± 9.3	70 ± 9.0	94	28	6	3	1949 ± 531	92 ± 20	3.4 ± 0.3
5	2	80	1	77.3	2.3	137 ± 9.6	66 ± 2.3	71 ± 1.5	130	56	6	2	12,226 ± 1411	203 ± 137	7.5 ± 0.7
6	2	57	1	96.2	2	136 ± 9.5	75 ± 3.6	60 ± 3.5	126	34	6	1	3988 ± 796	148 ± 44	4.2 ± 0.3
7	2	53	0	94.8	1.4	154 ± 5.7	87 ± 5.3	75 ± 7.6	96	50	2	0	15,811 ± 3026	31 ± 1	6.4 ± 1.7
8	2	68	0	83.2	2.3	165 ± 9.9	78 ± 8.6	74 ± 2.9	138	30	2	0	50,399 ± 5116	106 ± 10	5.1 ± 0.7
9	1	70	0	49	3.5	130 ± 13	73 ± 7.6	98 ± 14	96	56	3	0	15,657 ± 5218	103 ± 9	11.3 ± 3.8
10	1	57	2	115.3	1.7	155 ± 18	69 ± 3.8	88 ± 8.1	86	50	5	0	1868 ± 301	35 ± 0	3.1 ± 0.5
11	1	71	1	73.3	0	151 ± 9.6	74 ± 6.7	76 ± 2.6	94	30	3	0	4685 ± 378	27 ± 1	4.4 ± 1.1
12	1	78	0	60	1.8	170 ± 6.1	92 ± 7.5	81 ± 5.5	86	29	4	2	50,304 ± 4527	181 ± 20	20.8 ± 5.8
13	1	73	0	59.5	3.7	100 ± 6.8	67 ± 8.3	116 ± 4.7	108	20	2	0	70,000 ± 0	54 ± 4	5.0 ± 2.7
14	1	72	0	69.5	0	163 ± 5.3	73 ± 1.2	83 ± 3.5	86	60	3	1	4461 ± 1093	68 ± 6	7.9 ± 1.5
15	2	49	0	74.7	2.6	131 ± 8.5	71 ± 9.8	77 ± 1.7	102	20	4	2	13,438 ± 2068	67 ± 10	6.2 ± 0.7
16	1	42	0	92.9	0	165 ± 4.9	90 ± 5.7		98	56	2	0	590 ± 97	13 ± 2	2.7 ± 0.2
17	2	68	0	78	3.1	173 ± 5.7	81 ± 2.8	70 ± 8.5	104	56	5	1	24,108 ± 4227	83 ± 2	6.4 ± 1.2
18	2	68	0	92.2	0.9	187 ± 5.0	83 ± 3.2	48 ± 2.1	112	44	3	0	64,813 ± 8984	136 ± 7	7.2 ± 0.9
19	2	77	0	70	0.9	161 ± 21	70 ± 2.1	49 ± 1.0	92	56	4	2	2765 ± 167	41 ± 2	4.4 ± 0.4
20	2	59	2	115	1.6	120 ± 4.9	68 ± 19.8	73 ± 0.7	86	56	6	1	1221 ± 395	130 ± 14	5.9 ± 0.9

Gender: Women = 1, Men = 2; DM: Diabetes mellitus = 1, including nephropathy = 2; Dry weight: Body weight estimated normal after HD, BP=blood pressure, QRS: QRS duration in ECG, Charlson score: morbidity measure (score 0–30), CVD: Number of types of cardiovascular diseases (myocardial infarction, congestive heart failure, stroke, peripheral vascular disease); Patient code 13 is missing since the patient received a kidney transplant during the study and therefore not fulfilled all series.

### Study design

The design allowed each patient to be investigated during a mid-week HD session, in a randomized order. Previous studies showed that air exposure is different during various modes of HD^23,24,29,30^ and may deposit in the body^27,28^ and interfere with biocompatibility.^26^ Since frequently used synthetic dialyzers are made of polysulfone, we decided to use one commonly used product in Europe: the steam sterilized dry stored and compare with a gamma sterilized wet stored dialyzers that is more common in Japan. A reason for such choice was to clarify if such factors may partly explain that the prognosis of Japanese dialysis patients is much better than in Europe and USA.^16^

Each patient underwent three hemodialyses within the study, with one-week interval. Each HD with a different mode of low-flux polysulfone dialysis. Therefore all patients performed HD with three different modes: (a) a dry-stored low-flux dialyzer (F8HPS, Fresenius Medical Care, steam sterilized, polysulfone, surface area 1.8 sqm) with a lower blood level in the venous chamber (Dry-Low), (b) the same dialyzer but with a high blood level (Dry-High), and (c) a wet-stored low-flux dialyzer (Rexeed18L, Asahi Kasei Medical, gamma sterilized, polysulfone, surface area 1.8 sqm) with a high blood level (Wet-High). Standard HD was carried out using Fresenius (Bad Homburg, Germany) 4008/4008H/S devices in 56 sessions and 5008 in one session. The tubing sets used were Fresenius FA404C/FA404B steam sterilized. Gambro Lund AK 200S was used in three sessions. The tubing sets used were Gambro BL207B steam sterilized.

All patients were dialyzed by all three modes and were randomized to start and proceed with one or other HD mode. Each patient underwent one mid-week HD that was included in the study, with different modes, three consecutive weeks. Standard non-study dialyses were performed between the study dialyses. These standard dialyses were equal for each individual patient and considered as a wash out period of 1 week. The low-flux modes of dialyzers were used to minimize loss of laboratory markers by clearance through the membrane. Blood samples were drawn before HD and at 30 and 180 min of HD. All samples were collected from the arterial site of the dialysis circuit, before entrance into the dialyzer.

Samples collected for blood and plasma values of large molecules, cells and platelets were adjusted for the change in blood volume caused by fluid intake or fluid removed by ultrafiltration.^19^ Achieve adjusted (Adj) laboratory value (LabV) thereby was achieved using the laboratory value at timepoint T and dividing with the ratio change in blood hemoglobin^1^ at a specific time point (T) versus Hb at baseline (B):

$${\mathrm{LabV}}_{\mathrm{Adj}}={\mathrm{LabV}}_{T}/\left({\mathrm{Hb}}_{T}/{\mathrm{Hb}}_{B}\right)$$

The fluid retention between dialyses was estimated from interdialytic weight gain (IDWG) calculated as the percentage increase of body weight between two dialyses.^14,19^ A mean of the IDWG during one month was calculated and constituted the extent of ultrafiltration necessary to achieve an estimated dry weight.

Blood was collected in citrate and EDTA tubes and plasma was stored in -80^o^C. Each of the following variables was analyzed at the same time to avoid errors between series. Based on half-life, Pro-BNP (ref <125 ngL^−1^ for patients aged 0–74 and <450 ngL^−1^ for elderly, Roche) and high sensitive TnT (ref < 15 ngL^−1^, Roche) were measured before HD and at 180 min during HD. Differences between 180 min and before HD values were calculated (*delta-*values). Pro-BNP was occasionally raised above the upper limit of 70,000 ngL^−1^ (of the laboratory), for those samples the upper limit was set as 70,000 ngL^−1^. Changes above that limit were excluded from the statistical comparison of differences in Pro-BNP between baseline and 180 min of HD.

Analyses performed within 2 h of blood collection (Sysmex) were hemoglobin (Hb, ref 100–120 gL^−1^), erythrocyte volume fraction (EVF, ref 0.30–0.36), erythrocyte particle concentration (EPC, ref 3.6–4.2 × 10E12L^−1^), total leukocyte count (LeC, ref 3.5–8.8 × 10E9 L^−1^), neutrophil granulocytes (Neu, ref 1.8–6.3 × 10E9L^−1^), lymphocytes (Lym, ref 1.0–3.5 × 10E9L^−1^), monocytes (Mon, ref 0.3–1.2 × 10E9L^−1^), eosinophils (Eos, ref 0.07–0.3 × 10E9L^−1^), basophils (Bas, ref 0.0–0.1 × 10E9L^−1^), and thrombocytes (Thr, ref 145–348 × 10E9L^−1^). After plasma storage at −80°C analyses were performed of tissue plasminogen activator activity (TPAact, ref 0.2–2 IUmL^−1^), tissue plasminogen activator mass (TPAmass, ref 1–20 µgL^−1^), plasminogen activator inhibitor 1 mass (PAImass, ref 4–43 µgL^−1^), fibrin degradation product D-dimer (D-dimer, ref <0.20 mg L^−1^), von Willebrand factor (vWF, ref 206–238 U L^−1^), C-reactive protein (CRP, ref <5 mg L^−1^), pentraxin 3 (PTX, ref <3.5 µg L^−1^), activated complement factor 3, according to Ekdahl et al. (C3a, ref 20–130 µg L^−1^),^31^ thrombin antithrombin complex (TAT, ref <3 µg L^−1^), for C3(H_2_O), that is, full length complement factor 3 with a broken thiol ester (iC3), analyzed according to Ekdahl et al.^31^; there is a tentative reference value of 95–1015 µL^−1^ based on 20 healthy individuals.

The restriction of repeated numbers of samples such as at 30 and 180 min was based on the rapid activation caused by HD of some mechanisms such as the complement system and leukocyte and platelet depletion known to appear when using cellulose based dialyzers.^20^ Those factors are also known to normalize quite rapidly. Other factors are mainly expected to rise upon intervention and depend on metabolic half-life and removal by dialysis to accumulate during the 180 min of observation period, such as troponin T and Pro-BNP. The latest time to include all samples from a patient was at 180 min since patients ended HD sessions at different times.

Baseline cardiac conditions of the patients was assessed from the QRS duration (measured automatically from a routine 12 lead ECG) and ejection fraction (measured from a pre-dialysis echocardiogram or magnetic resonance function).^32,33^ In addition, the Charlson comorbidity score (grade 0–30, includes malignancy as variable)^34^ and a cardiovascular score (-score) based on the history of number of cardiovascular areas involved (myocardial infarction, chronic congestive heart failure, stroke, peripheral vascular disease) were calculated.

Microembolic signals (MES) were measured at the return bloodline, after the venous air trap, using ultrasound (CMD-10, Hatteland, Royken, Norway). Autopsy studies,^27,28^ showed that such signals at this point are caused by air emboli covered by fibrin or solely clots present in the blood returning to the patient. Cumulative data were achieved at 30 and 180 min.

### Statistical analysis

When planning the study, the number of patients included in the paired design were estimated based on alfa- and beta-error. Inclusion of 20 patients could reveal a significance if a requested effect was present in at least 70% of the patients with a risk of alfa-error of 5% and power of 80%, using G-power test.^35^ Data were analyzed using SPSS (PASW Statistics for Windows, Version 24. Chicago: SPSS Inc.). Wilcoxon signed rank test was used for paired samples, Mann-Whitney *U* test for independent samples. Correlations were analyzed with Pearson’s test (r-value) and the non-parametric Spearman’s test (rho-value). Values are expressed as median, mean ± standard deviation (SD) if not stated otherwise. Significant values were defined as a two-tailed *p*-value <0.05. The *p*-value should be put in its relation to the extent of secondary analyses, considering the Bonferroni concept of alfa-error by multiple analyses. Since patients were evenly distributed in all three modes of treatments the analyses were performed both for all aggregated dialyses (*n* = 60) and for each specific mode of HD (each *n* = 20). Multiple linear regression analyses were performed using the stepwise model and included two-tailed significant variables based on the Spearman’s test.

## Results

### Baseline values

Most samples of Pro-BNP (in 100%), TnT (in 96%), and PTX (in 78%) were higher than the upper normal limit (Table 1).

Baseline data related to cardiac structure and function were compared with all pre-HD values of Pro-BNP, TnT and pentraxin. Pro-BNP correlated with QRS-duration (rho = 0.37, *p* = 0.005), IDWG (rho = 0.54, *p* < 0.001) and age (rho = 0.30, *p* = 0.024) and inversely with the ejection fraction (rho = −0.45, *p* < 0.001); TnT with Charlson score (rho = 0.56, *p* < 0.001), -score (rho = 0.30, *p* = 0.023), IDWG (rho = 0.37, *p* = 0.005) and age (rho = 0.49, *p* < 0.001). The QRS duration correlated with the IDWG (rho = 0.35, *p* = 0.006). No univariate correlation was found with systolic or diastolic blood pressure, or heart rate. In multiple linear analysis of Pro-BNP as dependent factor the remaining significant variables were inverse ejection fraction, older age and higher IDWG (*r*^2^ = 0.63, *p* < 0.001). For TnT as dependent factor, Charlson score, -score and age remained significant (*r*^2^ = 0.72, *p* < 0.001). For pentraxin as dependent factor, age remained significant (*r*^2^ = 0.32, *p* = 0.013).

Patients with EF < 50% had higher Pro-BNP than those with EF ⩾ 50% (30059 ± 26381 vs 6870 ± 7555, *p* < 0.001) while TnT and PTX did not show such differences. Patients with QRS duration ⩽ 100 ms had lower Pro-BNP (10595 ± 14066 vs 28964 ± 27641, *p* = 0.010) and lower IDWG (1.5 ± 1.5 vs 2.7 ± 1.4, *p* = 0.004) compared to those with QRS > 100 ms.

### Impact of HD

**Figure 1.**
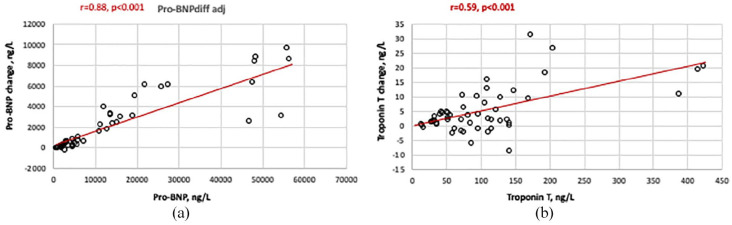
(a) Pro-BNP at baseline in relation to the change of Pro-BNP at 180 min of dialysis (correlation analysis by Pearson test). (b) Troponin T at baseline in relation to the change of Troponin T at 180 min of dialysis (correlation analysis by Pearson test).

**Figure 2.**
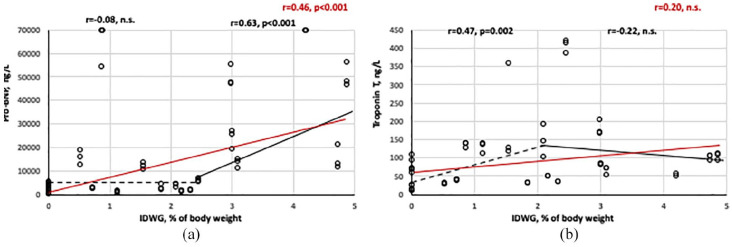
(a) Pro-BNP at baseline in relation to interdialytic weight gain (IDWG). The red line and *r*-value represents the whole material while the hatched line and *r*-value (above) represents IDWG ⩽ 2.5% and the full line represents IDWG values >2.5% (*r*-value above; correlation analysis by Pearson test). (b) Troponin T at baseline in relation to interdialytic weight gain (IDWG). The red line and *r*-value represents the whole material while the hatched line and *r*-value (above) represents IDWG ⩽ 2.5% and the full line represents IDWG values >2.5% (*r*-value above; n.s. = not significant; correlation analysis by Pearson test).

**Figure 3.**
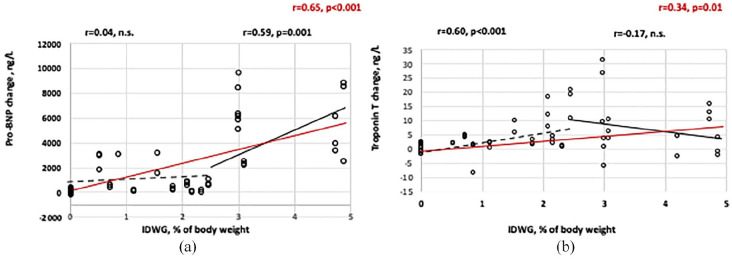
(a) Pro-BNP change from baseline to 180 min in relation to IDWG. The red line and *r*-value represents the whole material while the hatched line and *r*-value (above) represents IDWG ⩽ 2.5% and the full line represents IDWG values >2.5% (*r*-value above; correlation analysis by Pearson test). (b) Troponin T change from baseline at 180 min in relation to IDWG. The red line and *r*-value represents the whole material while the hatched line and *r*-value (above) represents IDWG ⩽ 2.5% and the full line represents IDWG values >2.5% (*r*-value above; n.s. = not significant; correlation analysis by Pearson test).

Table 2A displays mean and SD of baseline of various markers before HD and the differences after 30 and 180 min of HD, also analyzed for each separate mode of HD (Table 2B). Mean blood pump flow rate used was 317 ± 45 mL min^−1^ (Figure 1–3; Table 2).

**Table 2A.** Distribution of mean value and std. deviation (SD) and *p*-values for samples compared with base line data either at 30 or 180 min. Paired comparisons were made using Wilcoxon non-parametric method for all series together (ALL) or separately for series low-dry (low), high-dry (high), and wet high (wet high). Cumulative microembolic signals at 30 and 180 min of HD are given as MES.^a^.

Descriptive statistics	N	Base line data—all series		30 min data—all series		180 min data—all series		Difference start vs 30 min *p*-Values				Difference start vs 180 min p-Values			
		Mean	SD	Mean	SD	Mean	SD	All	Low	High	Wet high	All	Low	High	Wet high
MES^a^	60	0	0	1348	3096	6101	22,667	<0.001^b^	<0.001^b^	<0.001^b^	<0.001^b^	<0.001^b^	<0.001^b^	<0.001^b^	<0.001^b^
Pro-BNP	59	18,053	22,656	x	x	19,958	23,925					<0.001^b^	<0.001^b^	<0.001^b^	<0.001^b^
Troponin T	59	99	88	x	x	104	92					<0.001^b^	0.007^b^	0.014^b^	0.001^b^
Leukocytes, 10E9/L	60	6.6	2.3	6.1	2.1	6.5	2.5	<0.001^c^	0.004^c^	0.045^c^	NS	NS	NS	NS	NS
Thrombocytes, 10E9/L	60	234	102	224	96	225	93	<0.001^c^	0.006^c^	0.027^c^	0.003^c^	<0.001^c^	0.033^c^	0.037^c^	NS
Neutrophils, 10E9/L	56	4.2	1.8	4.2	1.7	4.4	2.1	0.038^c^	NS	NS	NS	NS	NS	NS	NS
Lymphocytes, 10E9/L	56	1.3	0.5	1.2	0.6	1.3	0.6	<0.001^c^	<0.001^c^	NS	NS	0.019^c^	NS	NS	NS
Monocytes, 10E9/L	56	0.58	0.21	0.46	0.17	0.54	0.19	<0.001^c^	0.001^c^	0.012^c^	0.006^c^	0.006^c^	0.035^c^	NS	NS
Eosinophils, 10E9/L	56	0.29	0.20	0.29	0.23	0.24	0.20	0.003^c^	0.012^c^	NS	NS	<0.001^c^	0.002^c^	0.005^c^	0.019^c^
Basophils, 10E9/L	56	0.05	0.02	0.04	0.02	0.04	0.02	0.004^c^	NS	0.036^c^	NS	<0.001^c^	0.011^c^	0.024^c^	0.026^c^
Pentraxin 3	60	6.4	4.3	7.8	5.0	9.7	5.5	<0.001^b^	<0.001^b^	<0.001^b^	<0.001^b^	<0.001^b^	<0.001^b^	<0.001^b^	<0.001^b^
PAImass	57	57	31	50	29	53	30	<0.001^c^	0.005^c^	0.005^c^	0.008^c^	0.003^c^	NS	NS	0.018^c^
TPAactivity	57	0.70	0.44	1.39	0.88	0.91	0.48	<0.001^b^	<0.001^b^	<0.001^b^	<0.001^b^	<0.001^b^	NS	0.001^b^	0.002^b^
TPAmass	57	8.4	6.0	11.2	9.0	8.3	6.1	<0.001^b^	<0.001^b^	<0.001^b^	0.001^b^	0.037^c^	NS	NS	NS
vWillebrand factor	57	289	157	293	146	311	161	NS	NS	NS	NS	NS	NS	NS	NS
CRP	57	5.2	9.8	5.3	10.2	5.0	10.4	NS	NS	NS	NS	NS	0.027^c^	NS	NS
D-dimer	57	767	821	718	695	739	739	<0.001^c^	0.002^c^	0.027^c^	0.02^c^	0.037^c^	NS	NS	NS
C3a	60	164	687	186	556	90^a^	33	<0.001^b^	0.001^b^	0.005^b^	0.004^b^	0.002^b^	0.005^b^	NS	NS
TAT	60	4.51	6.22	2.47	1.22	2.58	1.35	<0.001^c^	<0.001^c^	<0.001^c^	0.001^c^	<0.001^c^	NS	0.009^c^	<0.001^c^
C3(H_2_O)	60	8078	9118	12,126	31,620	6759	4169	NS	NS	NS	NS	NS	NS	NS	NS

a C3a median value at start 70, at 30 min 104 and at 180 min 82 units/L.

b Value is higher, NS = not significant (*p* ⩾ 0.05).

c Value is lower than baseline.

**Table 2B.** Cardiac markers: distribution of median, range (minimum-maximum value) and *p*-values for samples either at 30 or 180 min compared with base line data (Diff.). Paired comparisons were made using Wilcoxon non-parametric method.

Descriptive statistics	N	Base line data		30 min data		180 min data		*p*-Value diff. 30 min vs start	*p*-Value diff. 180 min vs start
		Median	Range	Median	Range	Median	Range		
All series									
Pro-BNP	57	5743	478–70,000	×	×	6490	482–70,000		<0.001^a^
Troponin T	57	81	12–422	×	×	104	13–443		<0.001^a^
Pentraxin 3	60	5.45	2.2–25	6.66	2.7–30	8.5	3.3–35	<0.001^a^	<0.001^a^
Low-dry dialysis mode									
Pro-BNP	18	5830	644–700	×	×	6576	693–70,000		<0.001^a^
Troponin T	18		13–386	×	×	79	13–397		0.007^a^
Pentraxin 3	20	6.1	2.6–22.6	6.7	3.2–28	9.1	4.0–35	<0.001^a^	<0.001^a^
High-dry dialysis mode									
Pro-BNP	19	5885	478–70,000	×	×	6995	482–70,000		<0.001^a^
Troponin T	19	84	12–414	×	×	85	13–433		0.014^a^
Pentraxin 3	20	5.5	2.6–25	6.8	2.9–30	8.3	3.4–31	<0.001^a^	<0.001^a^
High-wet dialysis mode									
Pro-BNP	20	5672	648–70,000	×	×	6221	659–70,000		<0.001^a^
Troponin T	20	71.5	15–422	×	×	77	14–443		0.001^a^
Pentraxin 3	20	4.8	2.2–14	6.1	2.7–18	8.1	3.3–19	<0.001^a^	<0.001^a^

a Value at 30 min or 180 min is higher than baseline value.

The effect of HD was analyzed by paired statistics: once comparing all dialyses baseline values with the change at either 30 or 180 min of HD and secondly similar analysis of each mode of HD. Most variables showed significant activation as a result of dialysis. Between the modes less activation of cells were seen for “high” blood level in the air trap and for “wet” dialyzer (Table 2A).

Hemodialysis resulted in raised cardiac biomarkers (*p* < 0.001) including PTX at 30 min (22%) and 180 min (53%), and at 180 min Pro-BNP (15%) and TnT (5%). There was similar increase in the cardiac marker between the dialyses modes, except for the rise in pentraxin at 180 min that was less in the Wet-High mode than in the Dry-Low (median 2.7 vs 2.8, *p* = 0.025, ending in 8.1 vs 9.1, *p* = 0.033, Table 2B).

The change of cardiac biomarker Pro-BNP from start to 180 min of HD correlated to the baseline levels of Pro-BNP (r = 0.88, *p* < 0.001; Figure 1(a)), as did the change of troponin T in relation to baseline TnT (r = 0.59, *p* < 0.001, Figure 1(b)) and similarly the change from start to 30 min of PTX (r = 0.28, *p* < 0.05).

There was a significant relation between IDWG and the baseline level of Pro-BNP with a significant breakpoint and rise for IDWG above 2.5% (Figure 2(a)). A significant relation between IDWG and baseline level of TnT was present during the first 2.5% of IDWG while it did not incline further thereafter (Figure 2(b)).

The change in Pro-BNP from baseline to 180 min was significant from a break point of an IDWG above 2.5% (Figure 3(a)). The change in TnT at 180 min was related to the IDWG up to a level of 2.5% of IDWG, thereafter not significantly changed (Figure 3(b)).

The HD-modes showed significant difference in MES count. Dry-Low had most, Dry-High less and Wet-High least counts: The median (min-max, Wilcoxon paired test) were at 30 min Dry-Low 1086 (92–7960), Dry-High 382 (34–22019, *p* = 0.028 vs Dry-Low), Wet-High 298 (0–2113, *p* = 0.002 vs Dry-Low) and at 180 min Dry-Low 4441 (174–30287), Dry-High 1603 (193–174710, *p* = 0.005 vs Dry-Low), Wet-High 878 (4–4838, *p* < 0.001 vs Dry-Low), respectively. Most MES signals/min appeared during the first 30 min of HD. When comparing the extent of change of variable in percentage from baseline between the three HD modes the lymphocytes were less lowered at 30 min when the Wet-High mode was used compared with the Dry-Low mode (−6.4 ± 16.6% vs −10.0 ± 16.4, *p* = 0.015) with similar impact at 180 min (−4.5 ± 16.6% vs −7.3 ± 21.7, *p* = 0.02).

Multiple regression analysis was performed with microembolic signals (MES) during the first 30 min of HD as dependent factor. No correlation existed when including all modes in analyses. For the mode Wet-High, a correlation existed with numerous variables resulting in an *r*^2^ of 1.0, shown in Table 3.

**Table 3.** Multiple regression analysis of the amount of microembolic signals (MES) measured at 30 min as the indirect dependent factor. The value 30 or 180 attached to a variable represents the change from baseline until either 30 or 180 min of HD. The values used for calculation were adjusted to the effect of ultrafiltration and the change from baseline given as percentage. The Table describes a stepwise model including only significant variables achieved for the dialysis mode wet-high.^a^

Model	*R*	*R* square	Adjusted *R* square	Std. error of the estimate	Change statistics		df1	df2	Sig. *F* change
					*R* square change	*F* change			
1	0.703^b^	0.494	0.458	471.318	0.494	13.665	1	14	0.002
2	0.862^c^	0.744	0.704	348.206	0.25	12.65	1	13	0.004
3	0.918^d^	0.843	0.804	283.315	0.1	7.637	1	12	0.017
4	0.956^e^	0.915	0.884	218.471	0.071	9.181	1	11	0.011
5	0.971^f^	0.943	0.915	186.842	0.029	5.039	1	10	0.049
6	0.985^g^	0.970	0.950	143.113	0.027	8.045	1	9	0.02
7	0.996^h^	0.992	0.986	76.835	0.022	23.223	1	8	0.001
8	0.999^i^	0.998	0.995	45.285	0.005	16.031	1	7	0.005
9	1.000^j^	0.999	0.998	27.258	0.002	13.321	1	6	0.011
10	1.000^k^	1	1	12.476	0.001	23.64	1	5	0.005
11	1.000^l^	1	1	7.514	0	9.785	1	4	0.035
12	1.000^m^	1	1	2.231	0	42.353	1	3	0.007
13	1.000^n^	1	1	0.449	0	72.191	1	2	0.014
14	1.000^o^	1	1	0.007	0	7183.286	1	1	0.008

Pred = predictors; C = constant; Sat = saturation; QB = blood pump speed is given as direct value (ml/min); TPAact = tissue plasminogen activator mass; PTX = pentraxin 3; PAImass = plasminogen activator inhibitor 1 mass; ProBNP = NT-Pro-BNP; iC3 = full length complement factor 3 with a broken thiol ester; vWF = von Willebrand factor; C3a = activated complement factor 3; Lym = lymphocytes, LeC = leukocytes.

a Setting = wet-high.

b Pred: (C), TPAact30.

c Pred: (C), TPAact30, PTX30.

d Pred: (C), TPAact30, PTX30, PAImass30.

e Pred: (C), TPAact30, PTX30, PAImass30, ProBNP180.

f Pred: (C), TPAact30, PTX30, PAImass30, ProBNP180, QB.

g Pred: (C), TPAact30, PTX30, PAImass30, ProBNP180, QB, iC330.

h Pred: (C), TPAact30, PTX30, PAImass30, ProBNP180, QB, iC330, Bas30.

i Pred: (C), TPAact30, PTX30, PAImass30, ProBNP180, QB, iC330, Bas30, vWF180.

j Pred: (C), TPAact30, PTX30, PAImass30, ProBNP180, QB, iC330, Bas30, vWF180, C3a180.

k Pred:(C), TPAact30, PTX30, PAImass30, ProBNP180, QB, iC330, Bas30, vWF180, C3a180, PAImass180.

l Pred: (C), TPAact30, PTX30, PAImass30, ProBNP180, QB, iC330, Bas30, vWF180, C3a180, PAImass180, Lym180.

m Pred: (C), TPAact30, PTX30, PAImass30, ProBNP180, QB, iC330, Bas30, vWF180, C3a180, PAImass180, Lym180, Sat15.

n Pred: (C), TPAact30, PTX30, PAImass30, ProBNP180, QB, iC330, Bas30, vWF180, C3a180, PAImass180, Lym180, Sat15, LeC180.

o Pred: (C), TPAact30, PTX30, PAImass30, ProBNP180, QB, iC330, Bas30, vWF180, C3a180, PAImass180, Lym180, Sat15, LeC180, Eos180.

As shown in Table 2A HD resulted in reduction of various types of leukocytes and platelets, and a rise in C3a, TPA-activity and TPAmass as measures of bioincompatibility between blood and the extracorporeal dialysis circuit and interaction with coagulation.

Blood pressure was measured during HD. No clinical hypotensive or hypertensive severe episode was registered during the study dialyses.

## Discussion

### Findings

The results of our analysis can be summarized as follows. *First*, after correcting for changes in blood concentrations by ultrafiltration, Pro-BNP and TnT significantly increased by 15% and 5%, respectively, after 180 min of low-flux HD, similarly for all three modes of low-flux HD. The similar outcome was present for the various modes of HD but varied depending on other factors than the setting. This has not been shown before, indicating a more general increase caused by dialysis per se in Pro-BNP and TnT. Such increase seemed consistent, and confirmed, what we have previously shown for low-flux HD,^19^ as have others reported for Pro-BNP.^17,18^ *Second*, the extent of rise in Pro-BNP and TnT was directly related to their baseline values and related to time on dialysis. This indicates that HD has a significant direct impact on myocardial function over time but in addition a more extensive strain is caused by HD especially in patients with existing impaired cardiac function. *Third*, HD resulted in significant increase in pentraxin 3, again relative to baseline value, and to the other cardiac biomarkers. *Fourth*, the rise in the three markers of cardiac function did, in principle, not differ between the three modes of HD. *Fifth*, IDWG seems to be an important factor to consider, not only in regard to the predialysis cardiac condition, but as a factor associated to cardiac strain during the dialysis procedure.

### Data interpretation

Our findings demonstrate a clear evidence for the HD impact on cardiac function, with three different markers of myocardial dysfunction showing significant rise. Pro-BNP represents cardiac endocrine function, secreted from the ventricles and the atria in response to increased wall stress,^36^ particularly in patients with stiff cavities such as cardiomyopathies and “significant diastolic dysfunction” known in uremic patients, irrespective of the commonly seen long standing hypertension. Due to its high sensitivity to changes in wall stress Pro-BNP is currently used as the main indicator in the clinical diagnosis of heart failure, irrespective of the presence of signs of decompensation. TnT, on the other hand, is secreted from the cardiac myocytes due to acute ischemic insults.

Since Pro-BNP is secreted in response to ventricular volume expansion and HD sessions ameliorate volume overload by removing excessive fluid, the observation that pro-BNP levels were raised by HD procedures seems paradoxical. However, we notice that the impact is worse when the volume overload (IDWG) is larger. This means that more extensive fluid shift by ultrafiltration could be the impact. Thereby effective dialysis (as fast artificial wash up) and more extensive ultrafiltration rate causes volume shifts and effects on the sub-endocardium that results in increased wall stress and release of Pro-BNP. A breakpoint for such stress seems to be at an IDWG above 2.5%. The TnT raise increases with the extent of IDWG to a limit of 2.5%. Thereafter it is kept constantly elevated. This was also found when comparing patients with mild versus significant left ventricular dysfunction in the present study.

Its perpetual leak with HD, also suggests direct myocardial injury, the explanation of which is likely related to the flow rates causing significant changes in loading conditions rather than the mere volume changes in the cardiac chambers, as is the case with Pro-BNP. Similar rises were also seen in pentraxin 3 despite its different mechanism of secretion. However, the lack of a relationship between pentraxin 3 and CRP negates the potential role of the inflammation pathway for its secretion but again strengthens the likelihood of the ischemic route or apoptosis suggested by others,^8^ including release of exosomes containing biomarkers.^37^ An additional impact on myocardial function was that of the altered saturation, we reported, which causes perpetual stiffness. Finally, the role of MES, constituting of air bubbles covered by fibrin, that we detected in our patients (at autopsy),^27,28^ could consequently worsen the subendocardial dysfunction and ischemia and hence a rise in the baseline markers’ levels. The microemboli passing from the dialysis device in to the vessels will mainly end up in the lungs, where they may contribute to a progressive fibrosis and pulmonary hypertension and subsequent cardiac strain.^27,28^ This would be strengthened by a majority of HD patients who suffer from pulmonary hypertension.^38,39^ Besides fluid overload the pulmonary hypertension will contribute to increased right heart work load what subsequently increases risk of mortality.^40,41^

Despite the numerous potential mechanisms for the secretion of the above three markers of cardiac function, our findings show that at 180 min of HD they all rose. In addition, the percentage increase was relative to their baseline levels, suggesting a close relationship with severity of myocardial dysfunction at control levels. Furthermore, the extent of rise of the three markers, in most cases, correlated with each other, particularly with the Wet-High dialyzing protocol thus demonstrating an evidence for a direct impact of HD on cardiac function irrespective of the underlying mechanism. A proof for the suggestion of multiple underlying pathophysiological mechanisms for the secretion of cardiac enzymes is supported by the different relationships with IDWG, with TnT the first to rise, followed by Pro-BNP, while pentraxin did not correlate with IDWG. Finally, the possible interaction between the air microemboli and the heart is indicated by multiple regression data of the Wet-High dialysis during the first 30 min exposure when also the change in Pro-BNP is a significant factor. However, even if air microemboli have a role they do not usually present as clinically evident symptoms. It seems unlikely that they can exert a biomarker effect in such a short period of time as studied here. Prolonged future studies are necessary in this field.

Our proposed interpretations are supported by findings of echocardiography *and* positron emission tomography studies which have shown that HD is associated with repetitive myocardial ischemia, in the absence of coronary artery disease, consistent with coronary microvascular dysfunction, pronounced fall in myocardial perfusion^42^ with an acute reduction in global and segmental myocardial blood flow and function.^43^ In addition, HD-induced myocardial stunning has also been reported,^44^ although the evidence behind is only modest, such change is not present in patients on peritoneal dialysis.^45^ The present study also showed that 180 min of HD even with synthetic membranes caused a significant stress of physiological mechanisms such as activation of the complement system, coagulation, cell activation and sequestration besides fluid shift by IDWG and exposure to microemboli of air. All factors that contribute to cardiovascular lesions.^14,21,27,28,46^

### Study limitations

(1) Although many patients in clinical practice undergo HD with high-flux membranes and HDF the study only included data performed by low-flux dialyzers. The reason was that we wanted to minimize loss of biochemical markers by clearance through the dialyzer. Therefore, the numeric results should not be simply applied to everyday clinical practice of HD, even if, according to Wahl et al.^17^, the mass balance of pro-BNP is greater in high-flux HD. Instead high-flux dialysis may give the false impression of improving cardiac factors.
(2) Some patients had Pro-BNP values above the cut off value of 70,000. Those data were not included in the statistical analysis of Pro-BNP changes, which reduced the sample size. Although the number of studied dialyses with each mode was limited the design (using patients as their own controls) allowed a high power and chance of finding differences.
(3) Side effects caused by microemboli may appear with a delayed effect and even after HD. It is not obvious that it will appear within the 180 min measurement period used in this study.
(4) Although both types of dialyzers were made of polysulfone and had surface area of 1.8 sqm, unknown differences may exist between the dialyzers used besides that F8HPS was sterilized by steam and Rexeed18L by gamma irradiation and stored wet.
(5) The end-point of the study was set at 180 min of HD to enable comparison of data from all patients. However, numerous patients performed longer sessions. The use of 180 min may well be a disadvantage since the physiological processes continue over time and outcome data could differ if analyses would have been performed after prolonged dialyses such as over-night. Besides the prolonged blood-membrane interaction also laboratory deviation of microbubble exposure might have been more obvious.
(6) The present study could not find multiple regression correlation between MES and the other variables found for Wet-High dialysis mode (Table 3) when analyzing the whole material or Dry-Low and Dry-High groups, nor when 180 min of MES was analyzed. The associations found for the Wet-High group only may indicate that the laboratory variables respond more linearly within a lower level of exposure to MES. Instead, when reaching a point above these levels the differentiation and correlation may be lost. Prolonged observation periods and less extensive exposure conditions in even larger studies may help clarify these results.

### Clinical implications

This study adds numerous new knowledges and clinical implications. Once we could notice a greater release of Pro-BNP during HD if high predialysis values, not only for Pro-BNP but also for TnT and pentraxin 3, were more increased. Frequent dry weight adjustment is necessary^47^ and IDWG is an important variable to measure. The risk with high IDWG have been emphasized before.^14,48–50^ In the present study it was possible to show a breakpoint of IDWG at approximately ⩽ 2.5% for cardiac strain during HD, marked by Pro-BNP. However, TnT rises also in those with lower levels during HD. The latter probably also as an indication of an inflammatory reaction. This prognostic level of less than 2.5% was suspected by outcome data in a previous study we performed.^14^

The present study supports that selection of various hemodialysis products should continue to focus on inducing minimal activation of biocompatibility in addition to optimizing the duration of the procedure. Technical improvements of the HD material are warranted both to reduce complement and coagulation reactions but also to reduce exposure of air to the patient. Intermittent chronic tissue damage by each HD session will add on to increase morbidity. The present study emphasizes importance of limited ultrafiltration in prolonged HD sessions, verified by release of cardiac markers as indication of cardiac stress if ultrafiltration is performed too rapidly. A worse prognosis by rapid ultrafiltration was also noted in a DOPPS study^51^ and also by shortened dialyses noted by experiences of the Tassin group.^52^ Since most patients have raised D-dimer, despite lack of clinical thromboses, this does not exclude resolution of microemboli. The benefits and strategies of anticoagulation have to be clarified in further studies.

## Conclusion

Hemodialysis, independent of type of dialyzer storage, was associated with raised cardiac biomarkers, more profoundly in patients with higher pre-dialysis values and especially during HD with larger fluid removal. A limitation in IDWG to < 2.5% and prolonged ultrafiltration time may limit cardiac strain during HD, especially in patients with cardiovascular risk.
